# Functional MRI Lateralization [M1] of dlPFC and Implications for Transcranial Magnetic Stimulation (TMS) Targeting

**DOI:** 10.3390/diagnostics13162690

**Published:** 2023-08-16

**Authors:** Jean Rama Surya, Barshen Habelhah, Jonathan Haroon, Kennedy Mahdavi, Kaya Jordan, Sergio Becerra, Victoria Venkatraman, Chloe Deveney, Alexander Bystritsky, Taylor Kuhn, Sheldon Jordan

**Affiliations:** 1 Neurological Associates—The Interventional Group, 2811 Wilshire Blvd #790, Santa Monica, CA 90403, USA; bhabelhah@gmail.com (B.H.); jharoon@theneuroassociates.com (J.H.); cdeveney@theneuroassociates.com (C.D.);; 2 Department of Psychiatry and Biobehavioral Sciences, UCLA School of Medicine, Le Conte Ave, Los Angeles, CA 10833, USA; 3 Department of Neurology, UCLA School of Medicine, Le Conte Ave, Los Angeles, CA 10833, USA

**Keywords:** functional lateralization, handedness, dorsolateral prefrontal cortex, language lateralization, functional MRI, task-based BOLD, repetitive transcranial magnetic stimulation, multimodal MRI

## Abstract

The present study investigates a potential method of optimizing effective strategies for the functional lateralization of the dorsolateral prefrontal cortex (dlPFC) while in a scanner. Effective hemisphere lateralization of the dlPFC is crucial for lowering the functional risks connected to specific interventions (such as neurosurgery and transcranial magnetic stimulation (TMS), as well as increasing the effectiveness of a given intervention by figuring out the optimal location. This task combines elements of creative problem solving, executive decision making based on an internal rule set, and working memory. A retrospective analysis was performed on a total of 58 unique participants (34 males, 24 females, Mage = 42.93 years, SDage = 16.38). Of these participants, 47 were classified as right-handed, 7 were classified as left-handed, and 4 were classified as ambidextrous, according to the Edinburgh Handedness Inventory. The imaging data were qualitatively judged by two trained, blinded investigators (neurologist and neuropsychologist) for dominant handedness (primary motor cortex) and dominant dorsolateral prefrontal cortex (dlPFC). The results demonstrated that 21.4% of right-handed individuals showed a dominant dlPFC localized to the right hemisphere rather than the assumed left, and 16.7% of left-handers were dominant in their left hemisphere. The task completed in the scanner might be an efficient method for localizing a potential dlPFC target for the purpose of brain stimulation (e.g., TMS), though further study replications are needed to extend and validate these findings.

## 1. Introduction

Handedness, or the propensity to utilize one hand more naturally than the other, is considered to be one of the more obvious functional asymmetries among humans. This asymmetry can be represented with neuroimaging techniques like blood oxygen-dependent level imaging (BOLD) [1]. Language lateralization in humans can be atypical depending on the participants’ lateralization of handedness; however, functional magnetic resonance imaging (fMRI) can be used to help determine laterality [2]. Less obvious functional activation asymmetries, such as language, creative problem-solving, and executive decision-making, require further investigation to determine neurological lateralization [3,4]. For example, language regions (e.g., Wernicke’s and Broca’s areas), as well as the dorsolateral prefrontal cortex (dlPFC), constitute key functional targets for various therapeutic interventions [5]. The dlPFC is known to be engaged with executive functions, such as working memory, decision making, creativity, and motor planning. The dlPFC is also functionally and reciprocally related to deeper prefrontal areas involved in mood regulation [6].

Hemispheric localization varies to some degree with handedness; however, there is no current “gold-standard” heuristic used to correlate handedness with language or dominant dlPFC lateralization, at least not with much certainty. There are, however, some observed trends that have been previously reported: among most right-handed individuals, dominant language function is most often localized to the left hemisphere. For many left-handed individuals, language can be lateralized to the left hemisphere as well; however, in contrast to right-handers, many left-handed participants show either a right hemisphere or bilateral representation [7]. Advanced neuroimaging could potentially help identify these patterns of activation and, therefore, improve individualized care.

The use of functional magnetic resonance imaging (fMRI) localization has already been proven to have utility in a variety of other medical conditions. The Wada technique, or the intracarotid sodium amobarbital procedure (ISAP), is known to be a ‘gold standard’ and important for physicians to establish hemispheric language lateralization and memory representation in presurgical evaluations for tumor and epilepsy procedures. Current research shows that functional MRI paradigms are being used to determine the localization and lateralization of language and memory centers in the brain [8,9]. Similar to how important this technique has been shown for surgeons to find the boundaries of surgical excisions, there is reason to believe that dlPFC lateralization matters for the determination of targeting neuromodulation, such as transcranial magnetic stimulation (TMS) treatments.

The effective hemispheric lateralization of the dlPFC is highly important, not only to decrease the functional risks associated with targeted interventions such as TMS but also to improve the efficacy of a given intervention by determining optimal placement. For example, repetitive transcranial magnetic stimulation has been shown to mitigate subgenual cingulate hyperactivity in participants with refractory depression, nearly toward the levels of healthy participants [10].

Additionally, previous research has suggested that the pre-treatment navigation of targets can both make the treatment less time-consuming, as well as improving patient outcomes [11]. However, most of the literature on the use of repetitive transcranial magnetic stimulation (rTMS) for patients with depression report a TMS application over the left dlPFC rather than the right, assuming that right-handers have a dominant dlPFC on the left hemisphere [12,13,14,15]. This prevalent assumption indicates that there may be a need to improve the level of unique, personalized dlPFC localization prior to treatments.

Therefore, this present study engages with a method to optimize effective functional lateralization strategies, particularly with regard to hemisphere asymmetry and dominance for the dlPFC. This task was designed to engage functional elements of creative problem-solving, executive decision making based on an internal rule set, and working memory, all during an MRI scan.

## 2. Materials and Methods

### 2.1. Participants

A retrospective analysis was performed on a total of 58 unique participants (34 males, 24 females, Mage = 42.93 years, SDage = 16.38) following consent to the IRB-approved protocol. Participants were recruited from a private-practice neurology clinic in Santa Monica, CA. Enrollment was conducted based on the number of participants undergoing concurrent TMS therapy; as such, the number of participants was not predetermined. Participants were excluded if unable to complete the fMRI scan; however, no participants met this exclusion criterion. Of these participants, 47 were classified as right-handed, 7 were classified as left-handed, and 4 were classified as ambidextrous. Handedness was classified using the Edinburgh Handedness Inventory, which was completed by all participants prior to MRI acquisition. The Edinburgh Handedness Inventory is the standard assessment of participant handedness and uses 20-Likert scale questions regarding which hand is used for specific tasks in order to determine which hand would be classified as ‘dominant’. A score of less than −100 to −61 classified participants as left-handed, while a score of +61 to +100 classified participants as right-handed. For a score between −66 and +40, participants were classified as ambidextrous [16].

### 2.2. Functional MRI Acquisition

MRI acquisition was performed on a 1.5 T Siemens Espree scanner equipped with a 16-channel head coil (Erlangen, Germany). A structural image was acquired first for co-registration with functional data. The T1 weighted anatomical sequence was magnetization-prepared with a rapid-acquisition gradient-echo (MPRAGE) (Repetition Time (TR) = 1810 ms; Time to Echo (TE) = 3.50 ms; Field of View (FoV) = 180 mm × 240 mm; resolution 1 mm isotropic). Each participant obtained blood oxygen level-dependent (BOLD) imaging at rest and with task-dependent activation. The resting-state bold images were 8 min and 20 s long. The resting state was acquired for another analysis that was not relevant to the activated task. It was not used to analyze this material. This functional sequence was acquired with a TR of 2500 ms, TE = 30 ms, FoV = 192 mm × 192 mm, resolution 4 mm isotropic, and 200 spatial volumes. The task-based BOLD acquisition was similar except for fewer volumes—a total of 140 brought the scan to 5 min and 50 s.

**Figure 1:**
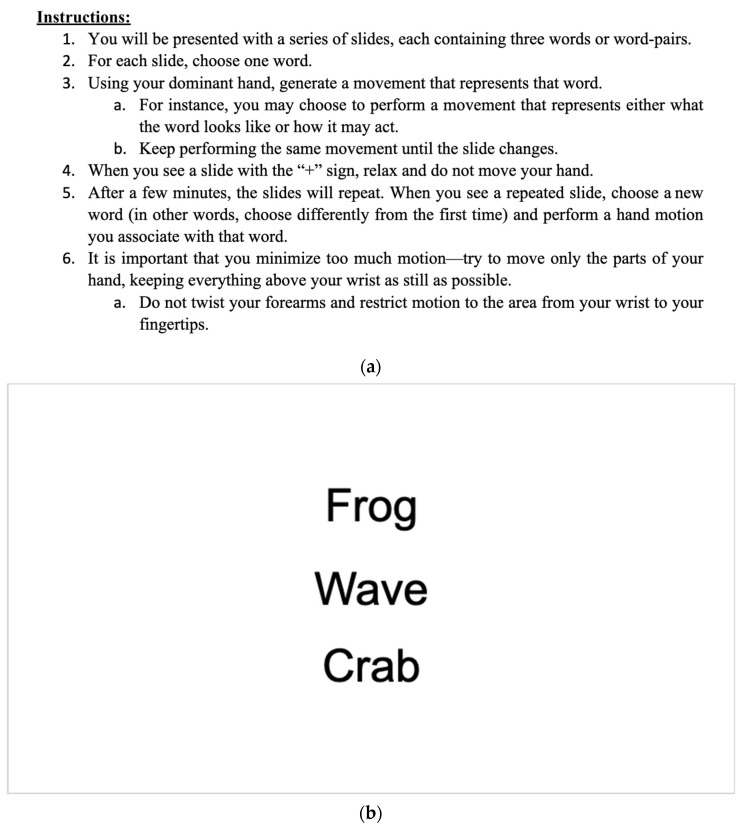

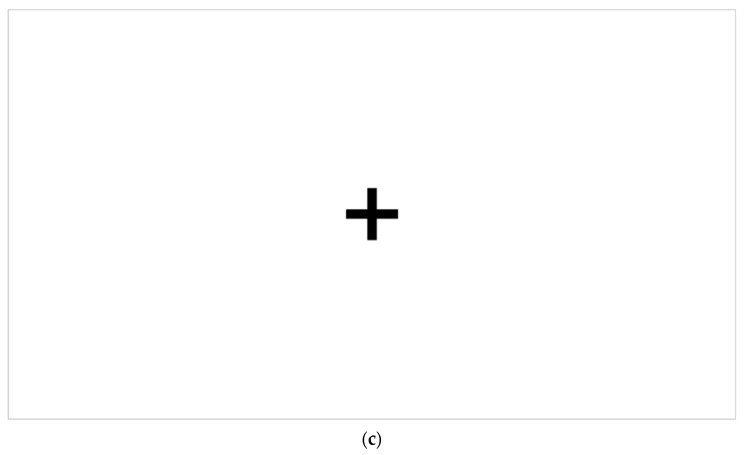

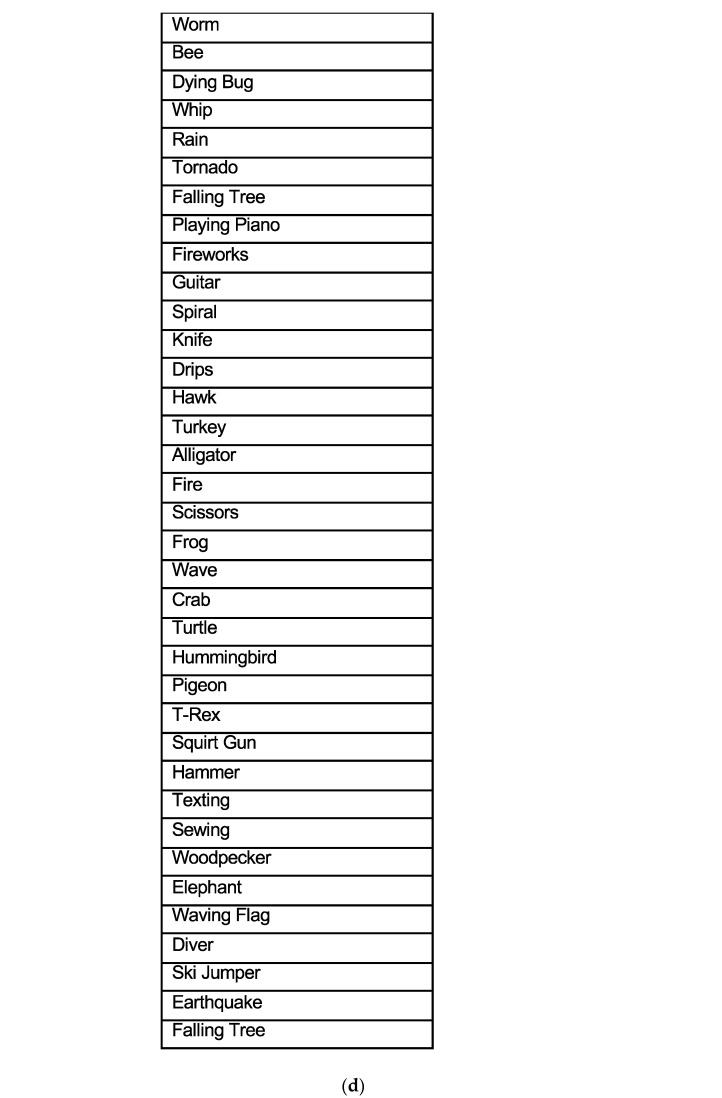
(**a**–**d**). Task-based instructions and example slides. (**a**). Instructions were given to participants before the fMRI scan. (**b**). Active task “On” slide. (**c**). Active task “Off” slide. (**d**) Table of words that were used during activated task sequence.

The active task performed during the BOLD sequence was designed to engage the dorsolateral prefrontal cortex (dlPFC) through participant decision making and creativity. Participants were trained by a technician before entering the scanner (Figure 1a) in the task’s nature. The technician presented the participant with these instructions and thoroughly walked them through the instructions line by line. The task was run using a block-based paradigm, constituting 25 s of rest (simply looking at a static “+” symbol centered on a screen (see Figure 1c) followed by 25 s of activity. During the scanning procedure, participants were shown a sequence of slides consisting of three words or word pairs. Each slide was displayed with the words centered on the screen, arranged in separate rows. The participants were then instructed to select one of the words and use their dominant hand to expressively depict the chosen word through a hand movement. This process is illustrated in Figure 1b.

To ensure an adequate sample of data, each stimulus block consisted of three slides, and each slide remained on the screen for a duration of 8 s with a brief pause between slides. In total, there were 7 stimulus blocks. The first four blocks were unique, while the last three blocks were repetitions of the first three blocks. During the last three blocks, the words from the first three blocks were repeated. The participant was instructed to choose a different word that they had seen and make a different hand movement (Figure 1a). The comprehensive list of words is listed below (Figure 1d). The words were chosen and reviewed by a focus group consisting of neurologists, neuropsychologists, and internal staff. This focus group of investigators determined over 100 words initially. To finalize the criteria, words were used that could be easily mimicked by hand and where there was no substantial duplication from one word to another in terms of mimic, distinct, and consensus. Part of the criteria was also making hand motions that were not extensive so that the participant did not move in the scanner. Before the scanning sequence was initiated, the technician reminded the participant about the task and then initiated the sequence. Participant cooperation was ensured during the task presentation by the MRI technician on site. Hand movements were monitored by the technician during the entire scanning sequence. If they failed to complete the task, the participant was reminded, and the scan sequence was restarted. Hand movements were not recorded as they were left up to the creativity of the participant.

This task aimed to actively engage several functions related to dlPFC processing. This included working memory and internally generated decision making. Participants internally selected the word they would invent a motion for, which elicited internally generated decision making. Their movement corresponding to the words encouraged working memory and creative engagement within a rule set. Working memory was required to choose a word that had not been previously demonstrated. Efforts were made to minimize excess motion by instructing participants to only move the dominant hand from the wrist to the fingertips; participants were encouraged to keep their forearms, elbows, and upper arms as still as possible while engaging in the active task. Participants were also encouraged to remain in a relaxed yet alert state throughout the exam.

### 2.3. Multimodal MRI Processing and Analysis

All participants received a multimodal MRI scan in order to specify dlPFC and motor cortex locations. Each multimodal MRI acquisition included anatomical 3D T1 whole brain MPRAGE imaging for detailed neuroanatomy as well as an fMRI co-registered network connectivity examination by resting state blood oxygen level-dependent (BOLD) imaging. The MRI data were acquired by the imaging center and sent to workstations for processing. Sub-investigators utilized the in vivo Dyna Suite Neuro software in our institute for processing imaging data. This software offers a range of automated functions to enhance the data. These functions include correction for head motion, spatial normalization to a standard brain, spatial smoothing, and statistical techniques that effectively isolate significant fMRI signal changes from noise.

During the data processing phase on workstations, there were several quality checks employed to ensure accuracy. These checks involved image alignment, co-registration, and skull stripping. Additionally, there were fMRI quality checks specifically focused on motion correction to guarantee reliable results. Multimodal MRI data processing methods employed through Dynasuite Invivo used a simple block design, with 25 s on, 25 s off, and spatial filtering set to 6 mm. A high-pass filter was used, and the block design was multiplied by a hemodynamic response function. Invivo Dynasuite included a motion correction check for quality assurance, ensuring that the translation was limited to <1.0 mm in x, y, z planes.

Computer scoring and human cross-checking were performed to ensure the adequate processing of artifacts, including proper co-registration and the appropriate selection of relevant data for final interpretation. A neurology and radiology peer review was performed on completed data sets for quality assurance and interpretation consensus.

The imaging data were judged by two blinded investigators for dominant handedness (primary motor cortex) and dominant dorsolateral prefrontal cortex (dlPFC). Criteria for lateral dlPFC activation were determined by the visual detection of a BOLD signal greater than or at 2.5 standard deviations from the mean in a prefrontal region located ≥5 cm anterior to the motor strip. Inter-rater reliability was defined by the joint probability of agreement from both investigators and was statistically assessed using Cohen’s Kappa interrater reliability coefficient [17].

### 2.4. Clinical Case Study

The present paper reports on the results of one participant among the 58 who presented with treatment-resistant major depressive disorder; they completed the multimodal MRI scan and subsequently underwent TMS treatment based on the results of dlPFC BOLD activation.

The TMS coil was targeted and placed on the participant’s dominant dlPFC based on findings from the lateralization of the fMRI. Parameters of TMS, when administered, included receiving 3000 pules at 10 Hz to their dominant hemisphere’s DLPFC, and motor thresholds were determined by a thumb twitch as in standard protocols. The participant was treated in 36 one-hour-long sessions, which adhered to typical rTMS guidelines. This participant also completed the Beck Depression Inventory (BDI) [18] and Beck Anxiety Inventory [19] upon being evaluated for TMS. These mood inventories were completed at the baseline and upon finishing treatment (within a week of their last session). Remission is defined as the completion BDI score < 12, BAI score < 14 [20].

## 3. Results

### 3.1. Overview

All 58 participants’ multimodal MRI scans were evaluated by two investigators who were blinded to the dominant handedness of the individual (primary motor cortex) and dominant dorsolateral prefrontal cortex (dlPFC). The agreement between Edinburgh Handedness and the activation pattern for which hand was utilized for the task was 100% (47 right-handed participants, 7 left-handed participants, and 4 ambidextrous participants).

The dorsolateral prefrontal cortex agreement between the two investigators who were blinded to the handedness of the individual was 87.9% (51 scans out of 58 scans), with a Cohen’s Kappa interrater reliability coefficient of 0.602, indicating a moderate–substantial agreement. The following analyses, therefore, were conducted using only the 51 scans, which had an agreement from both investigators.

Out of the 58 participants, there were 47 participants that were right-handed, for which the investigators agreed on the dominant dlPFC activation pattern of 42 of them. Of the 42 right-handed participants, 33 (78.6%) were left hemisphere dominant for the dorsolateral prefrontal cortex, and 9 participants (21.4%) were right hemisphere dominant for the dorsolateral prefrontal cortex.

Out of the total 58 participants, there were a total of seven left-handed individuals, and the investigators agreed on the dominant dlPFC of six participants. Of the six left-handed participants, five (83.3%) were right hemisphere dominant for dlPFC, and one (16.7%) was left hemisphere dominant.

**Figure 2:**
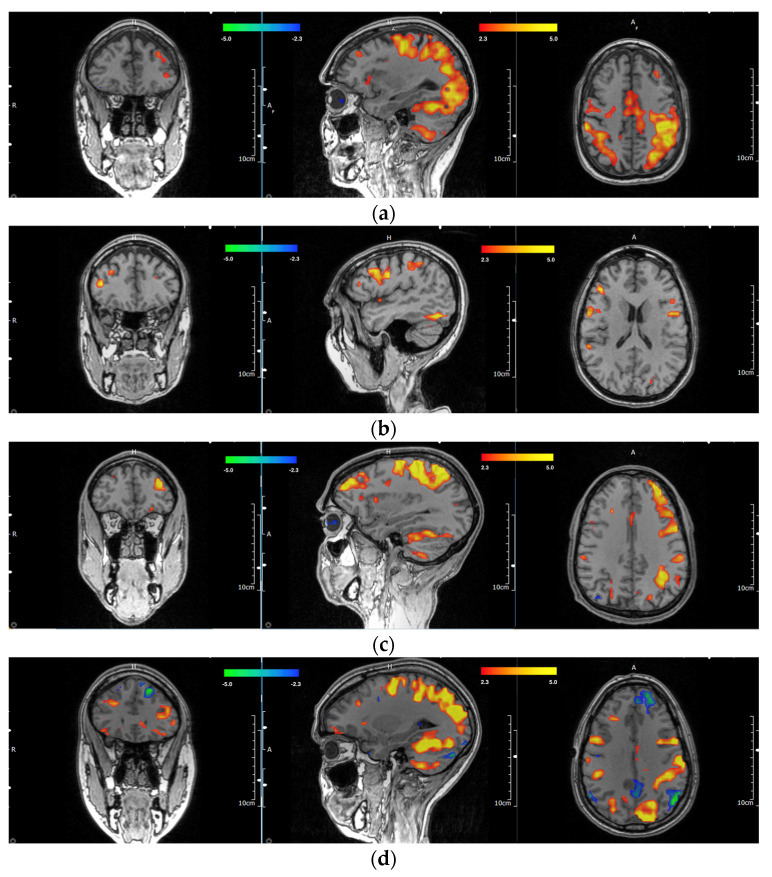

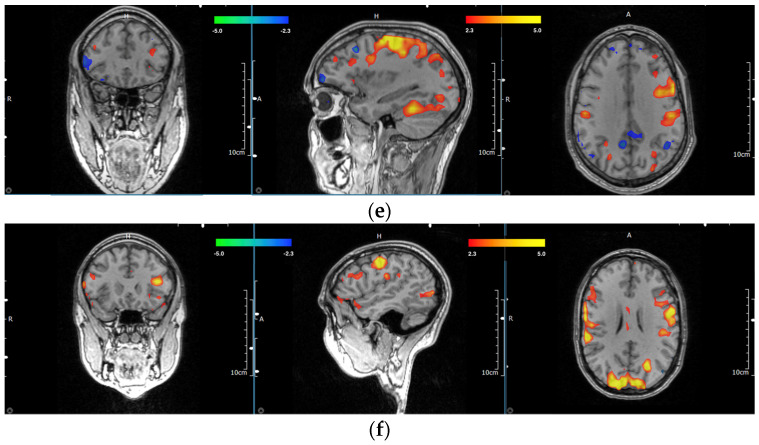
(**a**–**f**). Representations of different task-based handedness lateralization. (**a**). Coronal, sagittal, and axial sections of left-handed participant with left-sided dlPFC. (**b**). Coronal, sagittal, and axial sections of left-handed participant with right-sided dlPFC. (**c**). Coronal, sagittal, and axial sections of right-handed participant with left-sided dlPFC. (**d**). Coronal, sagittal, and axial sections of right-handed participant with right-sided dlPFC. (**e**). Coronal, sagittal, and axial sections of an ambidextrous participant with left-sided dlPFC. (**f**). Coronal, sagittal, and axial sections of an ambidextrous participant with right-sided dlPFC.

Out of the entire subset of 58 participants, there were a total of four ambidextrous participants, for which the investigators agreed on three of the scans for the primary dlPFC location. Of the ambidextrous participants, two (66.7%) were right hemisphere dominant, and one (33.3%) was left hemisphere dominant. Examples of multiple MRIs showing the handedness of individual participants with respective motor and dlPFC areas are shown in Figure 2a–f. A full breakdown of handedness and dlPFC lateralization can be found in Table 1. Full experimental details and results of all participants and their respective dlPFC scoring by blinded investigators can be found in Supplementary Table S1.

### 3.2. Case Study

Among the 58 participants imaged, one participant previously received TMS treatment at a different medical center prior to entering the clinical trial. The participant was stimulated on the assumed left hemisphere at the previous TMS center. However, this participant was right-handed and appeared to have a dominant dlPFC localized in the right hemisphere following fMRI scans using the present activation paradigm. At the initial TMS center, they were treated and stimulated in the assumed left hemisphere; consequently, the participant reported feeling worse both verbally and on several intake forms (e.g., Beck Depression Inventory and Beck Anxiety Inventory). The Initial Beck Depression Inventory (BDI) reported a score of 23, which was indicative of moderate depression. The participant’s initial Beck Anxiety Inventory (BAI) was noted to be at 13, which did not meet the criteria for a moderate–severe anxiety comorbidity. The participant then went into the imaging center to have the above-activated paradigm performed. After reviewing the participant’s scans and determining where the dominant dlPFC might be located, the participant underwent treatment again, targeted at the right dlPFC this time. The participant subsequently reported a decrease in the Beck Depression Inventory from 23 at pre-intervention to 10 post-intervention. This showed a 56.52% decrease, meeting the criteria for an improvement in depression status. The participant also reported a decrease in their Beck Anxiety Inventory score from 13 to 9, a 30.77% decrease.

## 4. Discussion

Out of the total population of 58 participants, on review of the multimodal MRI scans, the two investigators had a 100% agreement on which hand was used for the activation of these participants. They also had an 87.9% agreement on the dominant dorsolateral prefrontal cortex and agreed on 51 out of 58 scans on which dlPFC was activated during the scanning sequence. It is important to note that, in this retrospective analysis, the data showed that 21.4% of right-handed individuals showed a dominant dlPFC localized to the right hemisphere rather than the assumed left. This retrospective analysis also demonstrated that 16.7% of left-handers were dominant in their left hemisphere rather than the assumed right.

Potential reasons for why some participants may present with atypical dlPFC include gender differences, as well as the documented incidence of individuals who might have been born with the propensity for left-handedness but, for varying circumstances, were forced to become right-handed throughout their life [21,22]. Furthermore, another study that looked at prefrontal regions showed that there was lateralization depending on the age of participants [23].

Prior research in multiple imaging studies has suggested that depressed participants appear to have a decreased activation of the dorsal lateral prefrontal cortex with a reciprocal increased activation of the subgenual prefrontal region [24,25,26]. Both neurosurgical navigation and TMS targeting protocols have occasionally deployed seed analysis to determine stimulation sites or establish the bounds of a surgical field. For instance, a historical technique for localizing the dlPFC involves an anticorrelation analysis of a seed placed around the subgenual cingulate [27,28,29,30,31].

The case study participant previously discussed in the results was a right-handed individual who received TMS treatment before entering the clinical trial. This participant was treated in the clinic using assumed targeting to the conventional left dlPFC. After treatment, this participant reported feeling worse both verbally and on several intake forms (e.g., Beck Depression Inventory and Beck Anxiety Inventory). The participant underwent functional imaging techniques, and the blinded investigators reviewed the stimulated BOLD task. The investigators concluded that the participant’s dlPFC was dominant on the right side based on the criteria listed in the Material and Methods (Section 2.3). The participant then underwent treatment on the right side, with the results suggesting some benefit. This case study suggests that although participants are undergoing treatment based on their assumed contralateral hemisphere (depending on their lateralization of handedness), the dlPFC could, in fact, be lateralized on the ipsilateral hemisphere. This is an important finding as this shows that using an fMRI task-based paradigm can provide some insight into participants that can have a dlPFC that is dominant in the unassumed hemisphere. This could benefit participant treatment in TMS and, more specifically, when targeting the treatment of depression for participants.

Based on the current study, this might suggest that the effective lateralization of the dlPFC is important for improving the efficacy of stimulation techniques, such as repetitive transcranial magnetic stimulation, and for decreasing functional risks associated with neurosurgery. Tasks like the method used in this study with functional imaging could be one way for effective TMS targeting, as it allows for the participant to activate a region that encapsulates creative problem-solving, executive decision-making, and working memory.

Some limitations of this study were that the sample size could be larger and there could have been an additional blind investigator. As the field continues research in this area, there needs to be an improvement in the sample sizes and an increase in the database size of participants that have dlPFC localized to the non-traditional side. It would also be beneficial to report on the participants that the investigators did not agree on to show that it is even possible to have a contralateral crossover and bilateral dlPFC activation [32]. While this sample size could be increased in further studies, the task completed in the scanner seems to be an efficient way of localizing a potential dlPFC target for purposes of individual TMS neuronavigation.

As research grows in stimulation techniques, a suggestion for future research on this topic could include clinical correlations with task-based fMRI sequencing. This would entail correlating dlPFC activation with TMS targeting and treatment with clinical outcomes to see how effective dlPFC lateralization could be on participants that have dlPFC localized to non-traditional sides. This could prove widely effective for participants that are dominant in the dlPFC contralateral compared to traditional treatment sites. Clinically, this could be effective for participants as it could potentially show how targeting the correct dlPFC could lead to better clinical outcomes of treatment. There are trials currently attempting to further this line of research, as demonstrated in a TMS study that looked at a more personalized approach to TMS targeting for depression using multimodal and functional imaging techniques [33].

Future studies could expand on this research to delineate patterns of dlPFC lateralization among participants that have been trained to convert dominant handedness at a young age [34]. This could potentially explain why some right-handed individuals have a dominant dlPFC localized in their right hemisphere rather than their left. Further research regarding effective targeting is required to determine the most efficient way to localize brain regions that are responsible for creativity, problem-solving, and decision-making based on an internal rule set. Furthermore, this research could potentially be expanded using additional MRI sequences like arterial spin-labeling (ASL), diffusion tensor imaging (DTI), or task-based BOLD approaches to investigate areas of language lateralization.

In summary, with this fMRI task, their aim is to help determine dlPFC lateralization and subsequent implications for TMS targeting. Clinically, TMS targeting is usually assumed to be the treatment of depression using the dlPFC. It should be noted that task-based paradigms are vital to help correctly map targets for individualized participant treatment. This task-based sequence has been shown to help determine dlPFC lateralization and the dominant hemisphere as well as the dominant motor cortex. As this research expands, clinicians and researchers could work together to individualize participant care and treat participants more effectively using advanced imaging techniques, as discussed.

## Figures and Tables

**Table 1:** Representation by handedness and dlPFC lateralization with agreement by blinded reviewers (N = 51).

Handedness		DLPF Hemisphere	
Right Handed	42 (82.3%)	Right	9 (21.4%)
		Left	33 (78.6%)
Left Handed	6 (11.7%)	Right	5 (83.3%)
		Left	1 (16.7%)
Mixed Handed (Ambidextrous)	3 (6.0%)	Right	2 (66.7%%)
		Left	1 (33.3%)

## Data Availability

Data will be made available to qualified users upon formal request.

## Supplementary Materials

The following supporting information can be downloaded at: https://www.mdpi.com/article/10.3390/diagnostics13162690/s1, Table S1: Full breakdown of all participants and their respective demographics, handedness, and blinded reviewer assessment of dlPFC lateralization.
