# Supplementary material for: Functional MRI Lateralization [M1] of dlPFC and Implications for Transcranial Magnetic Stimulation (TMS) Targeting
Source: Diagnostics (Basel). 2023 Aug 16;13(16):2690. doi: 10.3390/diagnostics13162690 (PMC10453109; doi:10.3390/diagnostics13162690)
Supplement: Supplementary file 1 [file diagnostics-13-02690-s001.zip › diagnostics-2483071-supplementary.pdf]

Table S1. Full breakdown of all participants and their respective demographics, handedness, and blinded reviewer assessment of dlPFC lateralization.

Participant	Diagnosis	Gender	Age	Edinburgh Handedness	Blinded Reviewer #1	Blinded Reviewer #2
P1	Major Depressive Disorder (MDD), Pudendal Nerve Entrapment	M	36	RIGHT	LEFT	LEFT
P2	MDD	F	63	LEFT	LEFT	LEFT
P3	MDD	M	55	RIGHT	LEFT	LEFT
P4	MDD	M	72	RIGHT	LEFT	LEFT
P5	MDD, Lumbar Radiculopathy	F	62	RIGHT	LEFT	LEFT
P6	MDD	F	23	RIGHT	RIGHT	RIGHT
P7	MDD	M	49	RIGHT	LEFT	LEFT
P8	MDD	M	22	RIGHT	LEFT	LEFT
P9	MDD	M	26	RIGHT	RIGHT	LEFT
P10	MDD, Unspecified Sleep Disorder	M	26	RIGHT	RIGHT	RIGHT
P11	MDD	M	17	LEFT	RIGHT	RIGHT
P12	MDD, Migraine with Aura	F	39	RIGHT	LEFT	LEFT
P13	MDD, Post Traumatic Stress Disorder	M	20	AMBI	RIGHT	LEFT
P14	MDD, Obsessive Compulsive Disorder	M	50	RIGHT	LEFT	LEFT
P15	MDD, Headache	F	62	LEFT	LEFT	RIGHT
P16	MDD	F	23	RIGHT	LEFT	LEFT
P17	MDD	M	28	RIGHT	LEFT	LEFT
P18	MDD	F	56	RIGHT	LEFT	LEFT
P19	MDD	F	50	RIGHT	LEFT	LEFT
P20	MDD	M	63	RIGHT	LEFT	LEFT
P21	MDD, Chronic Migraine, Tinnitus	M	20	RIGHT	LEFT	LEFT
P22	MDD	F	48	AMBI	RIGHT	RIGHT
P23	MDD	M	26	RIGHT	RIGHT	RIGHT

P24	MDD	F	52	RIGHT	LEFT	LEFT
P25	MDD	M	45	RIGHT	LEFT	LEFT
P26	MDD, Thoracic Outlet Syndrome	F	62	RIGHT	LEFT	LEFT
P27	MDD	F	52	RIGHT	LEFT	LEFT
P28	MDD	F	21	RIGHT	LEFT	LEFT
P29	MDD	F	66	AMBI	RIGHT	RIGHT
P30	MDD, Myalgia	F	30	RIGHT	RIGHT	RIGHT
P31	MDD	F	40	RIGHT	LEFT	LEFT
P32	MDD, Fibromyaglia, Sleep Apnea	M	43	RIGHT	RIGHT	LEFT
P33	MDD, Lumbar Radiculopathy	M	73	RIGHT	RIGHT	RIGHT
P34	MDD, Traumatic Brain Injury	M	33	RIGHT	LEFT	LEFT
P35	MDD, Spastic Hemiplegia on Right Side	M	34	RIGHT	RIGHT	RIGHT
P36	MDD	M	51	RIGHT	LEFT	RIGHT
P37	MDD	M	75	RIGHT	RIGHT	LEFT
P38	MDD, Trigeminal Neuralgia	M	63	RIGHT	LEFT	LEFT
P39	MDD	F	36	RIGHT	LEFT	LEFT
P40	MDD, Lumbar Radiculopathy, Trigeminal Neuralgia	F	37	RIGHT	LEFT	LEFT
P41	MDD	M	24	RIGHT	RIGHT	RIGHT
P42	MDD, Dysuria	M	29	RIGHT	LEFT	LEFT
P43	MDD, Lumbar Radiculopathy	M	51	RIGHT	RIGHT	RIGHT
P44	MDD	F	55	LEFT	RIGHT	RIGHT
P45	MDD, Lumbar Radiculopathy, Spinal Stenosis	M	56	RIGHT	RIGHT	RIGHT
P46	MDD	M	29	RIGHT	LEFT	LEFT
P47	MDD, Fibromyaglia	F	36	LEFT	RIGHT	RIGHT
P48	MDD, Post Traumatic Stress Disorder,	M	39	AMBI	LEFT	LEFT

	Complex Regional Pain Syndrome					
P49	MDD, Tremor, Tinnitus, Lyme Disease, Encephalopathy	F	59	LEFT	RIGHT	RIGHT
P50	MDD	M	26	RIGHT	LEFT	LEFT
P51	MDD	M	62	RIGHT	LEFT	RIGHT
P52	MDD, Post Traumatic Stress Disorder, Migraine	F	36	RIGHT	LEFT	LEFT
P53	MDD	M	51	RIGHT	LEFT	LEFT
P54	MDD, Migraine	F	60	LEFT	RIGHT	RIGHT
P55	MDD, Idiopathic Epilepsy	M	59	RIGHT	LEFT	LEFT
P56	MDD	M	23	RIGHT	LEFT	LEFT
P57	MDD	F	24	RIGHT	LEFT	LEFT
P58	MDD, Generalized Anxiety Disorder	M	22	RIGHT	LEFT	LEFT
